# Draft Genome Sequences of Seven Strains of *Paenibacillus* spp. (Phylum *Firmicutes*) Inhabiting the Seeds of *Cucumis melo* L. (Cantaloupe) and Exhibiting Plant Probiotic Traits

**DOI:** 10.1128/MRA.00715-20

**Published:** 2020-08-20

**Authors:** Eman M. Khalaf, Manish N. Raizada

**Affiliations:** a Department of Plant Agriculture, University of Guelph, Guelph, Ontario, Canada; b Department of Microbiology and Immunology, Faculty of Pharmacy, Damanhour University, Damanhour, Egypt; University of Arizona

## Abstract

Here, we report the draft genome sequences of seven *Paenibacillus* sp. strains (EKM202P, EKM205P, EKM206P, EKM207P, EKM208P, EKM211P, and EKM212P) that were previously isolated from cultivated surface-sterilized seeds of *Cucumis melo* L. (cantaloupe). These candidate *Paenibacillus* plant probiotics displayed *in vitro* growth-promoting traits and suppressive activity against root-associated fungal/oomycete pathogens.

## ANNOUNCEMENT

In recent decades, the genus *Paenibacillus* has been described (1), and many strains are commercially exploited (2). Paenibacilli are recognized as plant symbionts, particularly root associated (2, 3); however, their sporulation capacity enables dormancy within seeds (4). High-throughput 16S rRNA gene sequencing showed that the genus *Paenibacillus* dominated cucurbit seeds, including melons (5), consistent with their cultivated microbiota (6). Seven unique bacterial colonies were selected from surface-sterilized seeds of *Cucumis melo* L. (cantaloupe), classified using the 16S primer pair 799F/1492R as *Paenibacillus* spp. (EKM202P, EKM205P, EKM206P, EKM207P, EKM208P, EKM211P, and EKM212P), and then submitted to GenBank (accession numbers KT281420.1, KT281430.1, KT281426.1, KT281421.1, KT281431.1, KT281427.1, and KT281423.1, respectively) (6). These microbes were assessed *in vitro* for their biostimulant/biocontrol potential. All strains demonstrated exoenzymatic activity (cellulase, pectinase, and protease [6] and RNase [7]) and acetoin/diacetyl (volatile) production and suppressed the plant pathogens Fusarium graminearum, Rhizoctonia solani, and Phytophthora capsici *in vitro* (7) (EKM212P was negative for pectinase [6] and RNase [7] activities). Exclusively, EKM202P and EKM212P suppressed Pythium aphanidermatum (7), while EKM202P and EKM205P grew on a nitrogen-free medium (LGI medium) (6).

With the use of −80°C glycerol stocks, the strains were cultured on LB agar. Single colonies were inoculated into lysogeny broth and incubated overnight at 37°C at 250 rpm. Genomic DNA was isolated from pellets using DNeasy UltraClean microbial kits (product number 12224-50; Qiagen) and then adjusted to 50 ng/μl. Libraries were constructed using TruSeq DNA Nano library preparation kits (KAPA HyperPrep kit KK8504) and then sequenced using the Illumina NovaSeq 6000 platform, which delivered 2,850,454 (EKM202P), 2,568,261 (EKM205P), 2,970,533 (EKM206P), 1,587,495 (EKM207P), 3,274,658 (EKM208P), 2,260,644 (EKM211P), and 2,754,662 (EKM212P) raw reads of the 150-bp paired-end format. *De novo* assembly of clean reads (quality score threshold, 30) was completed using the Evogene Clustering/Assembly Toolbox (EvoCAT) pipeline, and then the assembled contigs were taxonomically identified using KmerFinder v3.2 (8) leading to 115, 100, 118, 65, 136, 96, and 116-fold sequence coverage compared to that of *Paenibacillus* sp. strain M-152 (GenBank accession number NZ_CP034141.1) (EKM202P and EKM207P), Paenibacillus polymyxa strain YC0573 (NZ_CP017968.3) (9) (EKM205P and EKM206P), Paenibacillus polymyxa strain J (NZ_CP015423.1) (EKM208P and EKM212P), and Paenibacillus polymyxa strain HY96-2 (NZ_CP025957.1) (10) (EKM211P), respectively. Predicted proteins were defined using Prodigal (11) and matched against the NCBI nonredundant protein database using BLASTp (12), and then peptide domains were identified using InterProScan v5.32-71.0 (13). Assembly statistics and accession numbers are presented in Table 1.

**TABLE 1:** Characteristics and accession numbers of *Paenibacillus* genomes

Isolate	Bacterial species^*a*^	Genome size (bp)	No. of contigs	*N*_50_ (bp)	No. of genes	G+C content (%)	SRA accession no.	GenBank accession no.
EKM202P	*Paenibacillus* sp.	5,917,862	170	245,720	4,677	50	SRR11051668	JAAMNO000000000
EKM205P	*P. polymyxa*	5,731,588	208	460,107	4,600	52	SRR11051660	JAAMNU000000000
EKM206P	*P. polymyxa*	5,726,213	162	393,822	4,595	52	SRR11051647	JAAMNQ000000000
EKM207P	*Paenibacillus* sp.	5,907,745	170	204,300	4,682	49.5	SRR11051667	JAAMNP000000000
EKM208P	*P. polymyxa*	6,751,337	2,164	371,116	6,210	45	SRR11051674	JAAMNT000000000
EKM211P	*P. polymyxa*	5,764,313	119	221,247	4,529	48	SRR11051664	JAAMNS000000000
EKM212P	*P. polymyxa*	5,625,291	102	670,972	4,485	51.5	SRR11051658	JAAMNR000000000

a The taxonomy of these bacterial species is according to the updated GenBank databases.

All of the *Paenibacillus* genomes encode candidate proteins implicated in the aforementioned *in vitro* activities and additional traits, including tryptophan synthase (auxin production), carbon-nitrogen hydrolase, nitrogen regulatory protein PII, nitrogen assimilation/fixation (*nif*), phytase, alkaline phosphatase, and trehalose-6-phosphate hydrolase (2, 14, 15), cytokinin riboside 5'-monophosphate phosphoribohydrolase LOG (16, 17), and 1-aminocyclopropane-1-carboxylate synthase (growth/stress regulation) (18). Biocontrol/immunomodulation genes were identified for hydrolytic exoenzymes (chitinases, β-glucanases, lipases, proteases, pectin/pectate lyases, and ribonucleases) (2, 16, 19, 20), butanediol dehydrogenase-like enzymes (acetoin production) (1), iron siderophore-like compounds (aerobactin siderophore biosynthesis and IucA/IucC [exclusively EKM208P and EKM212P]) (2, 21), bacteriocins (thiopeptide type) (22), polyketide synthase and nonribosomal peptide synthase (lipopeptide synthesis) (15, 23), phenazine biosynthesis PhzF protein (except EKM211P) (24), alkyl hydroperoxide reductase (antioxidative enzyme) (25), and biomolecules for biofilm formation and quorum sensing (10, 14, 26, 27). This fundamental analysis may support future experiments to formulate new agricultural bioproducts.

### Data availability.

The whole-genome shotgun project and raw Illumina reads were deposited in DDBJ/EMBL/GenBank and the SRA, respectively, under the accession numbers provided in Table 1.
